# The Efficacy of a Web-Based Screening and Brief Intervention for Reducing Alcohol Consumption Among Japanese Problem Drinkers: Protocol for a Single-Blind Randomized Controlled Trial

**DOI:** 10.2196/10650

**Published:** 2018-05-30

**Authors:** Toshitaka Hamamura, Shinichiro Suganuma, Ayumi Takano, Toshihiko Matsumoto, Haruhiko Shimoyama

**Affiliations:** ^1^ Division of Clinical Psychology Department of Integrated Educational Sciences University of Tokyo Tokyo Japan; ^2^ Japan Society for the Promotion of Science Tokyo Japan; ^3^ Department of Psychiatric Nursing School of Medicine Yokohama City University Yokohama Japan; ^4^ Department of Drug Dependence Research National Institute of Mental Health National Center of Neurology and Psychiatry Tokyo Japan

**Keywords:** problem drinking, Web-based interventions, personalized normative feedback, Japanese drinkers, randomized controlled trial

## Abstract

**Background:**

The literature shows that computer-delivered interventions with personalized normative feedback can reduce problem drinking for up to 6 months in the West. Meanwhile, no studies have been conducted to examine the effects of such interventions among Japanese problem drinkers. Possible moderators associated with effectiveness of the intervention need to be also explored.

**Objective:**

The purpose of this study is to conduct a trial and examine the efficacy of a brief intervention with personal normative feedback and psychoeducation on several measures of alcohol consumption among Japanese problem drinkers. Additionally, this study will examine whether the level of alcohol use disorder and beliefs about the physical and psychological outcomes of drinking moderate the effect of the intervention on outcome measures.

**Methods:**

This study will conduct a single-blind, 2-armed randomized controlled trial. Japanese adults with an Alcohol Use Disorder Identification Test score of 8 or higher will be enrolled in the trial. Participants allocated to the intervention group will receive the intervention immediately after the baseline measurements, and participants allocated to the waitlist group will receive the intervention at the end of the trial. Outcome measures include drinking quantity, drinking frequency, and alcohol-related consequences. Follow-up assessment will take place at 1 month, 2 months, and 6 months following the baseline measurement. The authors will not know the group allocation during trial. The authors will plan to collect a sample of 600 participants. Mixed-effect analyses of variance will be used to examine the main effects of condition, the main effects of time, and the interaction effects between condition and time on outcome variables.

**Results:**

Enrollment for the trial began on January 6, 2018 and data are expected to be available by August 2018.

**Conclusions:**

This study will contribute to the literature by demonstrating the efficacy of Web-based screenings and brief interventions among Japanese problem drinkers and indicating several possible moderators between the intervention and outcomes. This type of Web-based brief intervention has the possibility of being implemented in Japanese schools and workplaces as a prevention tool.

**Trial Registration:**

UMIN Clinical Trials Registry R000034388; https://upload.umin.ac.jp/cgi-open-bin/ctr_e/ctr_view.cgi? recptno=R000034388 (Archived by WebCite at http://www.webcitation.org/6xmOoTfTI)

**Registered Report Identifier:**

RR1-10.2196/10650

## Introduction

### Background

Globally, alcohol use disorder is a common problem and the World Health Organization has reported that alcohol use is among the top five risk factors for disease, disability, and injury [1], resulting in 5.9% of all deaths [2]. According to a recent survey in Japan, alcohol abuse or dependence ranked as the most common individual disorder with a 7.4% lifetime prevalence, followed by major depressive disorder [3]. Although the Japanese government reports that average alcohol consumption has been decreasing since 1992 [4], 12.7% of males and 3.4% of females in Japan still engage in alcohol consumption of 60g or more, which is considered heavy drinking [3].

Furthermore, alcohol consumption by women has been increasing significantly, and men and women aged 40 to 59 years are particularly vulnerable to risky drinking [5]. However, the rate of individuals seeking and receiving professional help for alcohol-related problems is low. Surveys show that only 16.3% of Japanese individuals with substance use disorders received some form of treatment [6] and, crucially, only 1 in 50 (2%) of people with alcohol use disorders have sought professional help [7].

### Computer-delivered Interventions

One recent approach for treating alcohol-related problems is using computer information technology. Computer-delivered interventions have several advantages compared to face-to-face interventions with regard to the reduction of alcohol-related problems. Firstly, computer-delivered interventions have the advantage of user accessibility and the ability to reach a wide population via the internet. Second, computer-delivered interventions can minimize financial costs for the user. Although evidence is limited for alcohol-related problems, computer-delivered interventions have been shown to be relatively cost effective for other mental health problems, such as depression [8]. This approach can also reduce the burden on mental health care professionals. Lastly, the use of computer-delivered interventions reduces the stigma associated with treatments as some users prefer to receive interventions anonymously [9].

Recent studies have shown that internet interventions for alcohol-related problems can be beneficial [10,11], however, the effects of computer-delivered interventions are relatively limited. For example, several systematic reviews and meta-analyses show that computer-delivered screenings and brief interventions reduce alcohol consumption of the participants for up to 6 months, but the effects do not last longer than 12 months [12,13]. A meta-analysis shows multisession interventions have more sustained effects compared with single-session interventions [14], although one study reports no difference between the two types of interventions [15]. When compared to face-to-face interventions, computer-delivered interventions appear to be inferior [16].

### Personalized Normative Feedback

Many interventions that have shown the efficacy to reduce alcohol consumption include personalized normative feedback [17,18]. In personalized normative feedback, after users enter information about their demographics and alcohol consumption, they can compare their levels of drinking with the average levels of their age and sex.

Personalized normative feedback thus corrects users’ misperceptions of their level of drinking. Internet-based brief interventions with personalized normative feedback have been shown to reduce harmful alcohol use among Canadians [18], indigenous people in New Zealand [19], and Swiss young men [20]. Although effect sizes of these studies are between small and medium [18-20], a meta-analysis supports that personalized normative feedback is effective in reducing alcohol consumption [21].

Because both personalized normative feedback and psychoeducation involve cognitive restructuring regarding drinking patterns, further studies are needed to identify cognitive variables which can increase or decrease the effectiveness of the interventions. Alcohol expectancies are one variable to theoretically explain one’s drinking behavior. According to social learning theory, alcohol expectancies are individual beliefs about the effects of drinking on mood and behavior [22]. Alcohol expectancies are associated with frequency and quantity of alcohol consumption [23]. However, the current literature is limited in demonstrating whether the effectiveness of brief internet interventions differs according to the levels of alcohol expectancies.

In Japan, one study found that a psychoeducational video in a classroom setting increased knowledge of alcohol-related problems, but it did not reduce the number of alcohol-related problems among Japanese students in junior college at the 2-month follow up [24]. Although research in computer-delivered interventions has existed for about 20 years, very few computer-delivered interventions for reducing alcohol consumption exist in Japan. To our knowledge no prior studies have examined the effects of internet-delivered interventions using personal normative feedback among Japanese adults with problem drinking.

### Purpose of This Study

The first purpose of this study is to examine the efficacy of the Web-based intervention on reducing alcohol consumption among Japanese adults with problem drinking by conducting a pilot trial. To better understand the effects of the intervention, this study will also explore moderators between the intervention and outcome variables such as the degree of alcohol-related problems and participants’ beliefs about the effects of alcohol consumption (ie, alcohol expectancies). The authors hypothesize that a Web-based intervention will reduce the quantity and frequency of alcohol consumption among Japanese problem drinkers. Additionally, the authors hypothesize that variables such as alcohol-related problems and alcohol expectancies will moderate the effects of the intervention on outcome measures for alcohol consumption. The reduction of alcohol consumption will be greater among individuals with harmful and hazardous levels of drinking than those at the dependent level of drinking. A brief intervention will likely not suffice for individuals with a dependent level of drinking. Moreover, stronger negative or weaker positive beliefs about the effects of drinking will reduce participants’ alcohol consumption, due to participants’ belief that drinking will lead to negative consequences.

## Methods

### Description of the Screening and Brief Intervention (Your Health and Alcohol)

The authors have developed a Japanese Web-based screening and brief intervention based on a study conducted by Cunningham et al [18]. The development of the intervention took place between May 2017 and December 2018. This intervention consists of 3 sections: assessment, personalized normative feedback, and psychoeducation. The assessment section asks users to enter their demographic information such as age, sex, height, and weight, as well as average spending on one standard drink (see Multimedia Appendix 1). Users then enter their frequency of typical and heaviest alcohol consumption, the quantity of typical and heaviest alcohol consumption, and different areas of their lives affected by problem drinking over the past 12 months. These measures are partly taken from the Alcohol Use Disorder Identification Test (AUDIT) [25] and the Daily Drinking Questionnaire (DDQ) [26].

The personal normative feedback section of the intervention shows the users’ reported drinking level during a typical week, drinking level when they drank the most, frequency of heavy drinking (5 or more drinks in a day), and their AUDIT score. Pie charts and bar graphs are used to compare their levels of alcohol consumption with other individuals of their age and sex (see Multimedia Appendix 2). Estimated annual drinks, the cost of drinking, caloric intake, and areas affected by drinking are also provided. All these results are based on the assessment taken in the first part of the intervention. The reports of other individuals’ drinking patterns that users compare with their own were obtained from a previous survey collected from the community using a research marketing company.

The last part of the intervention is psychoeducation about the consequences of problem drinking. This section aims to educate users regarding the recommended amount of drinks (20g per day) according to a report from the Japanese government published by the Ministry of Health, Labor, and Welfare [27]. The process of digesting alcohol and the time it takes to break down alcohol is also provided in this section; based on the weight reported in the assessment section of the intervention, the website calculates the estimated time the user needs to break down consumed alcohol in the body. The website also informs the users about possible physical consequences (eg, damage to the liver, stomach, pancreas, circulatory system, hormones, and brain), psychological consequences (eg, aggression) and social consequences of excessive drinking (eg, increased risk of domestic violence). Information on ways to prevent and reduce problem drinking are also provided for users. Finally, this section includes multiple-choice questions to check how well users understand the psychoeducation materials (see Multimedia Appendix 3).

### Trial Setting, Recruitment, and Eligibility Criteria

All participation in the trial occurs online. Recruitment will take place through 2 crowdsourcing websites and 1 research marketing company. The authors used this online recruitment method since the population receiving this intervention are likely to be frequent internet users. Individuals registered on the systems will be asked to participate in the study through the posted URL. The URL will direct participants to a website created specifically for this trial. The inclusion criteria for participation is scoring 8 or higher on AUDIT [25] and being aged 20 years or over.

### Procedure and Allocation

Once participants are directed to the trial website, they will first take the screening measures (ie, AUDIT), and only those who meet the eligibility criteria will be invited to formally participate in this trial. Participants who do not meet the eligibility will be directed to a webpage showing that they are not eligible to participate in the study, whereas those who meet the criteria proceed to the next section without being told the screening results. Participants will be asked to spend between 5 and 15 minutes entering their typical drinking patterns and to think about their results and alcohol-related health. After participants fully read and understand the nature of this study, and consent to participate in the trial, they will be asked to enter their email address or their account name on the crowdsourcing site to receive notifications for subsequent follow-ups. They will then provide their demographic information and complete the measures of alcohol expectancies.

This study will be a 2-armed randomized controlled trial. Participants will be randomly allocated to either the intervention group or the control group. The website created for this trial automatically allocates participants to either group using computer-generated numbers. Both groups will complete the assessment part of the intervention. Only the intervention group will receive the personalized normative feedback and psychoeducation immediately after the baseline measures. The control group is the waitlist group of participants who will be notified that they will receive the intervention at the end of the participation.

**Figure 1:**
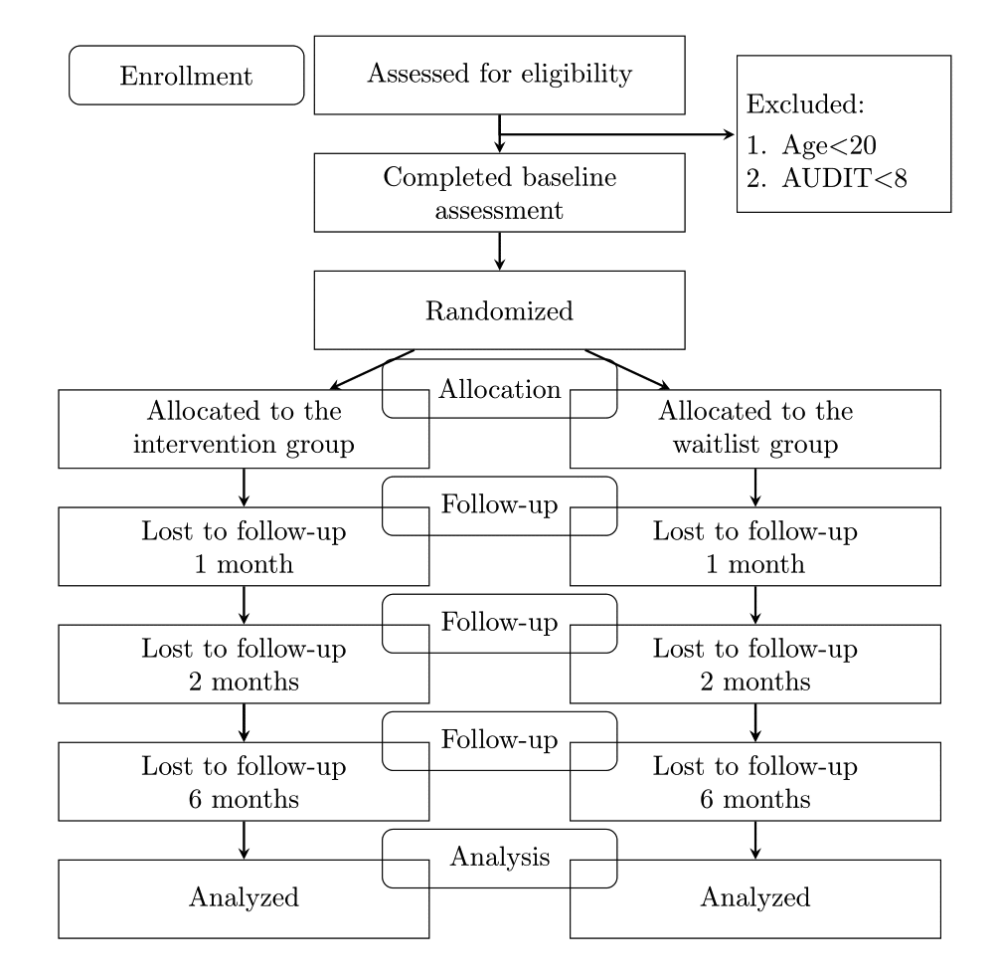
A CONSORT flowchart for the trial.

Figure 1 shows the flow of the trial. Participants will complete the outcome measures at baseline, 1 month, 2 months, and 6 months into the study. At each follow-up, participants will be asked to enter information about their drinking patterns since their last assessment. Specifically, they will be asked about their drinking patterns during the past month at the 1-month and 2-month follow-ups and their drinking patterns over the past 4-months at the 6-months follow-up (due to the previous assessments being conducted 1 and 4 months prior respectively). At the end of the trial participation, the participants will be thoroughly debriefed about the nature of the study. Participants registered in the research marketing company will be compensated with ¥120 (equivalent to US $1) and 1000 credits. Participants on the crowdsourcing website will receive ¥1200 (equivalent to US $11) as work compensation after completing all the follow-up measures.

### Interventions

This study will use the Web-based intervention (Your Health and Alcohol) developed prior to the trial (see above for the description of the intervention).

### Measures

Primary outcomes are the quantity of weekly alcohol consumption, quantity of largest alcohol consumption on one occasion, and frequency of drinking. The secondary outcome is the different areas of life which their drinking has affected. These measures are partly taken from the AUDIT [25,28] and DDQ [26] questionnaires and they have been previously used in other trials [18-20].

To investigate possible moderators between the intervention and outcome measures, the participant’s level of alcohol-related problems will be categorized as hazardous, harmful, and dependent according to their levels of AUDIT score [29,30]. Another measure is alcohol expectancies. In this measure, alcohol expectancies are defined as beliefs about physical and psychological effects of alcohol consumption [31]. Positive aspects of alcohol expectancies are mood enhancement and stress coping, and negative aspects of alcohol expectancies are physical ailments and dysphoria.

### Participant Timeline

Enrolment for this trial began on January 6, 2018.

### Blinding

At the beginning of the study, participants are informed that they will receive the intervention either immediately after the baseline or at the end of the trial. Meanwhile, all instructions to participants are uniform, and the authors will not know the participants’ allocation during trial.

### Ethical Procedures and Possible Harm

The research ethics board at the first author’s university approved the current study (approval #17-174). All procedures are according to the UMIN Clinical Trial Registry (R000034388) and follow the guidelines of Standard Protocol Items: Recommendation for Interventional Trials [32]. The authors anticipate the possible harm of this intervention is minimal because participants will simply receive information related to alcohol consumption. If participants experience distress and require professional assistance during the trial, the authors will refer them to appropriate treatment.

### Power Analysis

Previous studies have suggested small effect size for internet-based interventions for drinking consumption (1 drink less per week) [12]. A power analysis was conducted in R (software for statistical computing) and found that to obtain statistically significant results a total sample of 393 is necessary for 80% statistical power at *P*<.05 for significance level. One trial conducted in Japan reported a 65% dropout rate [33]. Because a high attrition rate is expected in internet-based research, the authors will collect a sample 600 of participants.

### Data Monitoring, Auditing, and Management

Since the intervention is a single session immediately subsequent to the baseline, the authors have determined that monitoring of participants’ reporting or interim analyses to continue or discontinue with the trial is not necessary. The authors do not plan to ask a third-party to audit the trial. All consent forms and private or identifying information will be separated during data analyses to protect anonymity.

### Statistical Analyses

To evaluate the feasibility of the website intervention, the authors will review participants’ comments about the intervention. Additionally, the authors will calculate the attrition rate from the enrollment to the final assessment. All data analysis will be performed in R [34]. First, descriptive statistics will be calculated to show the mean and standard deviation of measured variables. Data will be screened for missingness, outliers, linearity, normality, and homoscedasticity. Appropriate data transformation will be used for subsequent data analyses. Intention-to-treat analyses will be conducted to examine the efficacy of the intervention on all outcome measures. Missing data will be replaced with multiple imputations.

Linear mixed-effect model analyses of variance (ANOVA) will be used to examine the main effects of condition, the main effects of time, and the interaction effects between condition and time of the intervention on outcome measures. To examine the moderating variables between the intervention and outcome measures, alcohol expectancies and levels of AUDIT will be separately added into the model. Interactions of alcohol expectancies or AUDIT with condition, time, and condition × time (multiplying the two) will be analyzed. An R package called “nlme” will be used for these analyses [35].

## Results

Recruitment began on January 6, 2018, and data are expected to be available for analyses by November 2018.

### Dissemination Policy

Once data collection and analysis are completed, the authors will prepare a manuscript to publish the results in an academic journal. The authors plan to disclose the protocol or the results in first author’s academic thesis, academic conferences, and the journal in which the manuscript will be published.

## Discussion

### Overview

The purpose of this study is to conduct a single-blind 2-armed pilot randomized controlled trial to examine the efficacy of the website intervention for 6 months for reducing alcohol consumption among Japanese problem drinkers. This study will collect data using two different crowdsourcing websites and one research marketing company. Instructions are given uniformly throughout the trial, and thus the authors will not know the allocation of the participants. Data collection is expected to end in late 2018. The authors hypothesize that the intervention will have significant effects with a medium or small effect size consistent with a similar previous study [18-20]. The effect size will likely decrease over time but may last up to 6 months, since the literature shows that the effects of computer-delivered interventions do not last for longer than 6 months [12,13,36]. The authors hypothesize that alcohol expectancies will moderate the effects of the intervention on the outcome measures. Additionally, the authors expect that the levels of the AUDIT scores will moderate the effects of the intervention on the outcomes. Participants at the harmful and hazardous level of the AUDIT score will show larger effects from the intervention than those at the dependent level. These individuals with the dependent level will require more extended internet or face-to-face interventions.

### Limitations

This study comes with several limitations. The first limitation is accuracy of item responses. Converting alcohol consumption to a standard measure of drink will be an unfamiliar task for many Japanese individuals, and they may not report their AUDIT or the screening items accurately. For a second limitation, a sampling bias may exist because this study will recruit only individuals who are registered in a specific research marketing company or participating crowdsourcing websites. This sample may have different characteristics from those in the general population in Japan. Many of them are likely freelance workers working independently as opposed to belonging to a company or organization. This demographic difference may affect results of this study in terms of drinking as opportunities for social engagement. Social drinking is a common practice in Japan, and results of this study may not fully capture this drinking pattern.

Additionally, literature shows that internet-based studies commonly encounter high drop-out rates and inattentive responses during trial [33,37]. When a substantial portion of participants drop out of the study, this study can only reveal results of those who adhere to the intervention. This study is unable to reveal effects of the intervention among participants who discontinue with the study.

### Implications of the Study and Suggestions for Future Studies

As previous studies have revealed that seeking alcohol treatment is unpopular in Japan [3], computer-delivered interventions will play an important role for reducing alcohol consumption. To our knowledge this is the first study to examine the efficacy of an internet intervention with personalized normative feedback among Japanese problem drinkers. The authors argue that a Web-based screening and brief intervention can be useful to implement in various settings such as university or workforce. Since the legal drinking age is 20 in Japan, many university students are at risk of underage drinking and thus alcohol-related problems. This study excluded individuals engaging in underage drinking because of ethical responsibility. However, future studies may examine the efficacy of the intervention among university students by including students engaging in underage drinking. One study reports more than half of the students engaging in underage drinking in Japan [38]. Also, statistics show that in Japan men aged between 40 and 60 have the highest alcohol consumption compared to men in other ages or women [5]. Interventions with personalized normative feedback and psychoeducation can be used as a preventative tool before students or workers develop serious problems.

The literature shows ample evidence that alcohol expectancies are associated with drinking behavior [39]. Examining alcohol expectancies as possible moderators between effects of interventions and drinking behavior can contribute to the literature by applying theoretical understanding of drinking patterns into clinical settings.

Although more smartphone-delivered interventions have been used in recent approaches, the present study utilized a laptop or desktop computer. Users are likely in a less distracted environment when sitting in front of a laptop or desktop. Also, users can check the report from the intervention easily with a wider screen. However, future studies may develop a brief intervention using a mobile device and take advantage of its supporting functions.

The literature indicates that single-session interventions have limited effects on alcohol-related problems [40]. Future studies need to develop more rigorous interventions which improve the magnitude and duration of the effects. Previous studies have shown that extended multiple interventions have sustained effect sizes compared with single-session interventions [14]. Although this study offers only a brief single-session intervention, extended and theory-based computer-delivered interventions would provide added benefits for reducing alcohol-related problems.

## Appendix

Multimedia Appendix 1 Assessment in the Intervention.

Multimedia Appendix 2 Personalized Normative Feedback in the Intervention.

Multimedia Appendix 3 Psychoeducation in the Intervention.

## Abbreviations

AUDIT: Alcohol Use Disorder Identification Test
DDQ: Daily Drinking Questionnaire
