# Supplementary figures and images for: The Efficacy of a Web-Based Screening and Brief Intervention for Reducing Alcohol Consumption Among Japanese Problem Drinkers: Protocol for a Single-Blind Randomized Controlled Trial
Source: JMIR Res Protoc. 2018 May 30;7(5):e10650. doi: 10.2196/10650 (PMC6000480; doi:10.2196/10650)

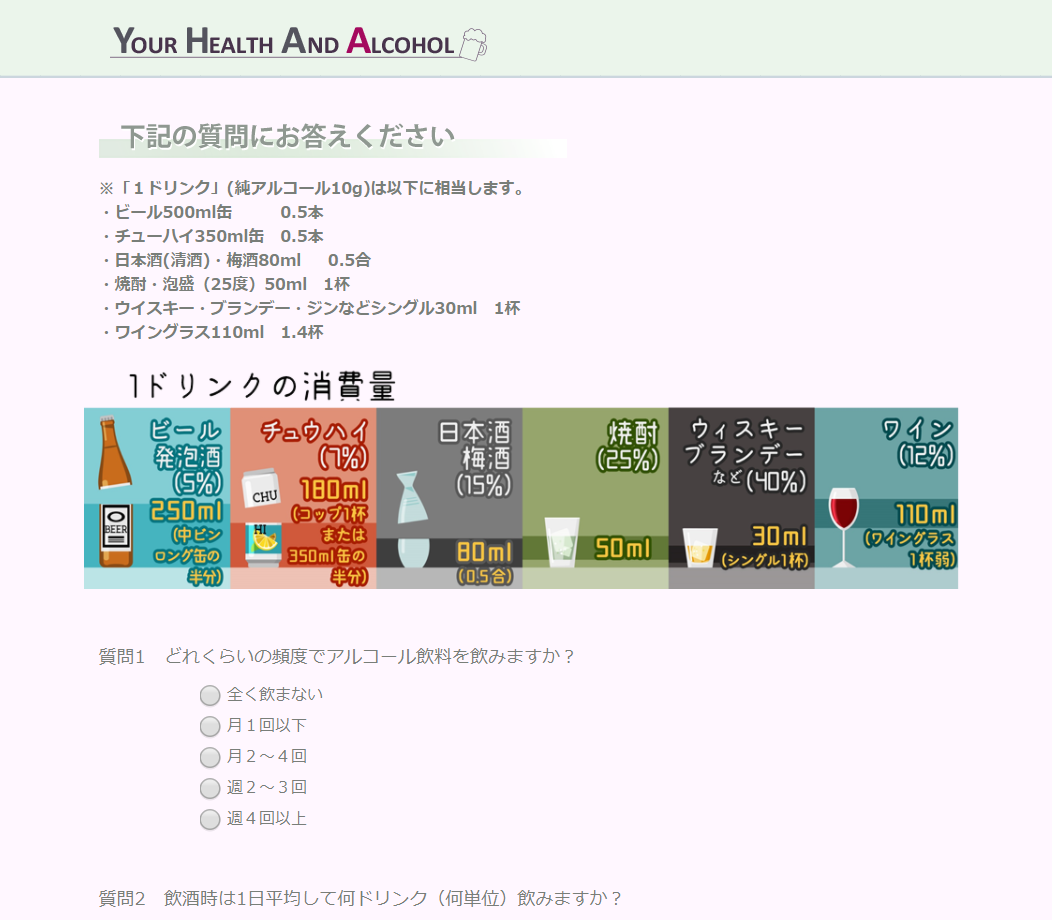

Supplement: Multimedia Appendix 1 [file resprot_v7i5e10650_app1.png]

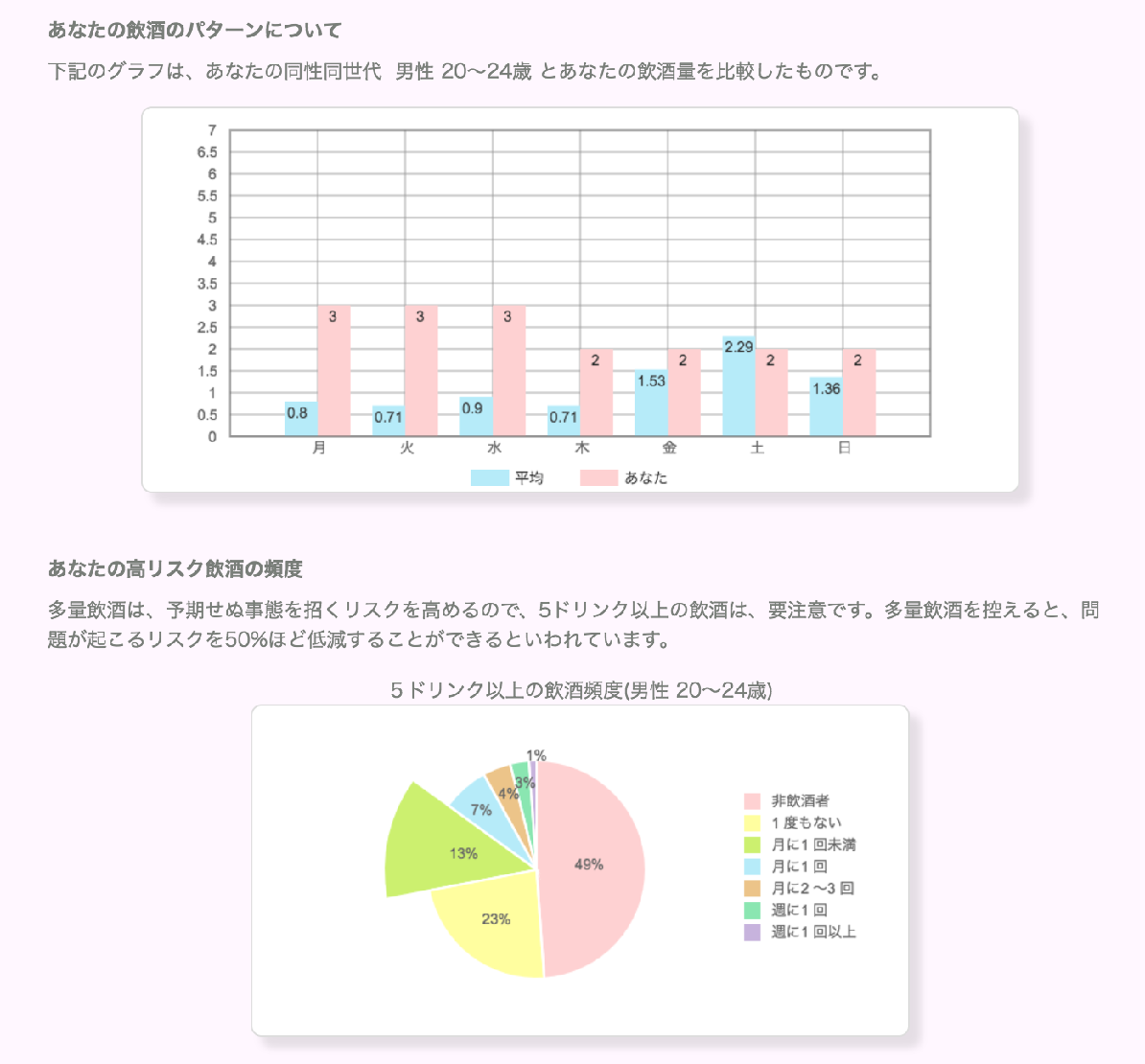

Supplement: Multimedia Appendix 2 [file resprot_v7i5e10650_app2.png]

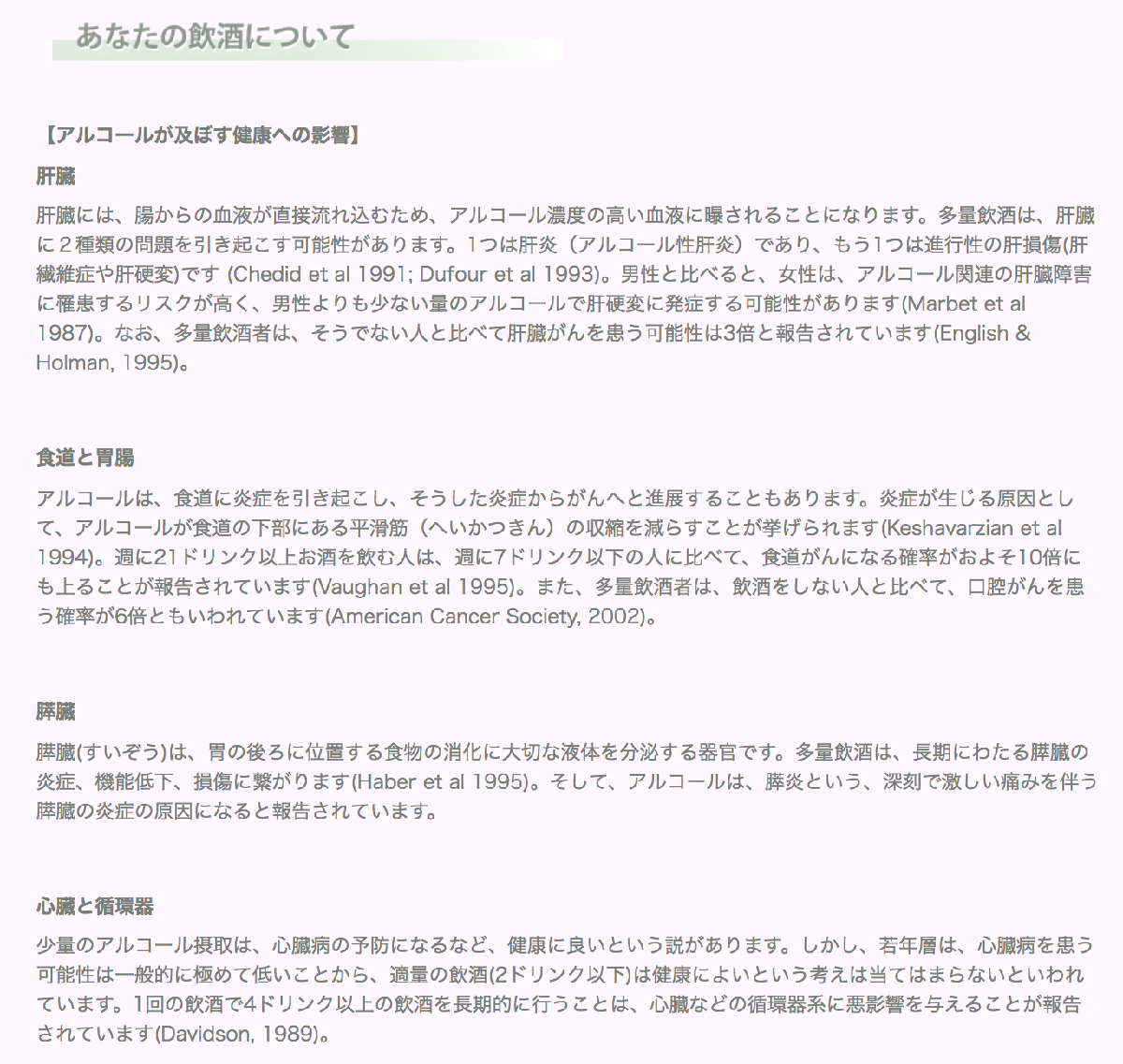

Supplement: Multimedia Appendix 3 [file resprot_v7i5e10650_app3.png]
